# Are Borrmann’s Types of Advanced Gastric Cancer Distinct Clinicopathological and Molecular Entities? A Western Study

**DOI:** 10.3390/cancers13123081

**Published:** 2021-06-21

**Authors:** Cristina Díaz del Arco, Luis Ortega Medina, Lourdes Estrada Muñoz, Elena Molina Roldán, M. Ángeles Cerón Nieto, Soledad García Gómez de las Heras, M. Jesús Fernández Aceñero

**Affiliations:** 1 Department of Surgical Pathology, Psychiatry and Pathology, School of Medicine, Complutense University of Madrid, 28040 Madrid, Spain; luis.ortega@salud.madrid.org (L.O.M.); jmariajesus.fernandez@salud.madrid.org (M.J.F.A.); 2 Department of Pathology, Hospital Clínico San Carlos, 28040 Madrid, Spain; elenamilagrosa.molina@salud.madrid.org (E.M.R.); m.delosangeles.ceron@salud.madrid.org (M.Á.C.N.); 3 Department of Pathology, Hospital Rey Juan Carlos, Móstoles, 28933 Madrid, Spain; lourdes.estrada@hospitalreyjuancarlos.es; 4 Department of Basic Medical Sciences, School of Medicine, Rey Juan Carlos University, Móstoles, 28933 Madrid, Spain; soledad.garcia@urjc.es

**Keywords:** gastric cancer, Borrmann classification, clinicopathological, prognosis, molecular, gross morphology

## Abstract

**Simple Summary:**

Borrmann classification is widely used for advanced gastric cancer (GC). Most studies on the clinicopathological impact of this classification have been performed in Asian countries, and almost all authors analyzed only type IV tumors. We assessed the clinicopathological, molecular features and the prognostic value of Borrmann types in all patients with advanced GC resected in a Western institution (*n* = 260). We observed a significant relationship between Borrmann types and several clinicopathological and molecular features, including age at diagnosis, systemic symptoms, tumor size, Laurén subtype, presence of signet-ring cells, infiltrative pattern, high grade, necrosis, size of the largest lymph node metastasis, HERCEPTEST positivity, microsatellite instability and molecular subtypes. No association was found between Borrmann classification and prognosis. According to our results, Borrmann types may represent distinct clinicopathological and biological entities. Further studies should be performed to confirm the role of Borrmann classification in the stratification of patients with advanced GC.

**Abstract:**

Most studies on the clinicopathological impact of Borrmann classification for gastric cancer (GC) have been performed in Asian patients with type IV tumors, and immunohistochemical features of Borrmann types have scarcely been analyzed. We assessed the clinicopathological, molecular features and prognostic value of Borrmann types in all patients with advanced GC resected in a Western institution (*n* = 260). We observed a significant relationship between Borrmann types and age, systemic symptoms, tumor size, Laurén subtype, presence of signet-ring cells, infiltrative growth, high grade, tumor necrosis, HERCEPTEST positivity, microsatellite instability (MSI) and molecular subtypes. Polypoid GC showed systemic symptoms, intestinal-type histology, low grade, expansive growth and HERCEPTEST positivity. Fungating GC occurred in symptomatic older patients. It presented intestinal-type histology, infiltrative growth and necrosis. Ulcerated GC showed smaller size, intestinal-type histology, high grade and infiltrative growth. Most polypoid and ulcerated tumors were stable-p53-not overexpressed or microsatellite unstable. Flat lesions were high-grade diffuse tumors with no MSI, and occurred in younger and less symptomatic patients. No association was found between Borrmann classification and prognosis. According to our results, Borrmann types may represent distinct clinicopathological and biological entities. Further research should be conducted to confirm the role of Borrmann classification in the stratification of patients with advanced GC.

## 1. Introduction

Gastric cancer (GC) is the fifth most frequent tumor worldwide and the third most common cause of cancer-related deaths [1]. Despite recent technological advances, five-year survival rates are estimated to be as low as 30% in Western countries [2].

GC is considered a complex and heterogeneous disease with varying etiology, incidence, prognosis and management [3,4]. It can be classified according to diverse features: location, gross morphology, microscopy or molecular alterations [5]. In recent years, several molecular classifications have been proposed, including The Cancer Genome Atlas and the Asian Cancer Research Group (ACRG) systems [6,7]. However, these classifications have not yet been translated into clinical practice [8,9,10].

On the other hand, Borrmann classification is a widely implemented and cost-effective classification that divides advanced GC into four types depending on its gross appearance: polypoid (type I), fungating (type II), ulcerated (type III) and flat/diffusely infiltrative (type IV) [11]. It has been variably related to patient prognosis, and clinicopathological features of Borrmann types have been mainly studied in type IV tumors [12,13]. Notably, almost all these studies have been performed in Asian countries. GC shows high geographical variation: Western and Asian areas present different epidemiological, diagnostic, prognostic and treatment features [2]. Therefore, until these differences decrease, the impact of the prognostic factors and clinicopathological features of GC should not be directly translated from Asian to Western populations, and Western studies should be conducted to characterize GC in low incidence countries. Unfortunately, Western studies are scarce.

As previously mentioned, GC is a heterogeneous disease and despite recent technological advances the only curative treatment is surgery: advanced tumors are treated by total or subtotal gastrectomy with lymphadenectomy. However, resected patients in Western countries still show poor survival rates and treatment options after surgery have not significantly changed in recent years. Several authors have highlighted the need to improve the stratification of GC patients for clinical trials and/or patient management, in order to find groups of patients who would benefit from different treatment options [8,9,10]. Molecular classifications are complex and expensive, so other investigators have analyzed the new roles of “classical” and cost-effective histologic features, such as the Laurén classification, which may reflect the biological heterogeneity of GC [8,9]. The identification of clinicopathological and molecular differences between Borrmann types may add another tool to stratify patients with resected GC. In this study, our aims were to assess (1) the clinicopathological and molecular differences between Bormann types and (2) the prognostic impact of Borrmann classification in Western patients with advanced GC resected with curative intent. As far as we know, no other study has characterized the clinicopathological and molecular features of type I–IV advanced GC in Western populations.

## 2. Materials and Methods

This study was a retrospective study including all patients undergoing surgery for GC at a tertiary referral center in Madrid (Spain) between 2001 and 2019. Patients were treated by total or subtotal gastrectomy with D1 or D2 lymphadenectomy. The investigators reviewed clinical records and collected demographic, clinical and follow-up data. Assessed variables included patient age, sex, symptoms at diagnosis, drinking and smoking habits, treatment, tumor progression and cause of death. Gross findings such as tumor size, tumor location and macroscopic type were taken from the database of the Surgical Pathology Department (PatWin).

### 2.1. Inclusion Criteria

A total of 377 GCs were resected in our institution between 2001 and 2019. After data collection, patients receiving neoadjuvant therapy, patients with metastatic tumors at diagnosis, patients with R1 or R2 resections and patients with T1 tumors were excluded from the study. Final analyses included 260 patients with advanced GC.

### 2.2. Histopathological Study

Specimens were formalin-fixed and paraffin-embedded. Slides were reviewed by two independent pathologists. The investigators followed a detailed protocol for histologic evaluation, and discordant cases were conjointly reviewed. Microscopic features of the tumors were assessed, including tumor type (Laurén classification), histologic grade, presence of signet-ring cells, perineural infiltration, lymphovascular invasion, tumor budding, desmoplasia, tumor necrosis, growth pattern (expansive or infiltrative), surgical margins, tumor extension (T stage), number of LN dissected, number of involved LN, lymph node ratio (LNR) and size of the largest lymph node metastasis. The LNR was computed as the ratio between the number of metastatic LNs and the total number of LNs retrieved from the surgical resection specimen. All cases were staged according to the 8th edition of the AJCC-TNM classification of tumors [14].

### 2.3. Immunohistochemical Study

Five tissue microarrays (TMAs), including a subgroup of cases from this cohort (*n* = 180), were constructed for immunohistochemical (IHC) study. TMA blocks contained two cores per case (tumor center and leading edge). The TMAs were assembled using the MTA-1 tissue arrayer (Beecher Instruments, Sun Prairie, WI, USA). Cores (diameter: 1 mm) were punched from pre-selected tumor regions in paraffin-embedded tissues. Then, 2 μm sections from the TMA blocks were obtained for IHC staining. Slides were deparaffinised by incubation at 60 °C for 10 min and incubated with PT-Link (Dako, Denmark) for 20 min at 95 °C in a high pH buffered solution. Holders were incubated with peroxidase blocking reagent (Dako, Denmark). Biopsies were incubated with the primary antibodies for 20 min, followed by incubation with the appropriate anti-Ig horseradish peroxidase-conjugated polymer (EnVision, Dako, Denmark) to detect antigen–antibody reactions. Sections were then visualized with 3,3′-diaminobenzidine as a chromogen for 5 min and counterstained with haematoxylin. Sections of the TMA block were immunostained for HERCEPTEST, p53, E-cadherin, MLH1, PMS2, MSH2 and MSH6 (all antibodies prediluted; Dako, Denmark) as previously stated. We included positive and negative controls for the IHC reactions. HERCEPTEST was evaluated according to the CAP recommendations [15]. GC cases were divided into molecular subgroups depending on the classification of the ACRG, as we described in a previous study [16].

### 2.4. Statistical Analysis

All data were stored in an anonymized Excel file and analyzed using IBM SPSS statistical package for Windows, version 20. Qualitative variables are presented using percentages and frequencies. Quantitative variables are described as arithmetic means with standard deviation (SD) or as the median and range, as appropriate. For the analysis of the association between variables, we used the chi-squared test for qualitative data and Student’s t-test to compare means between dichotomic quantitative variables. Statistical significance was settled at a *p*-value < 0.05. Overall survival (OS) and disease-free survival (DFS) curves were estimated with the Kaplan–Meier method, and significance was tested with the log-rank test. Multivariate analysis was performed using Cox regression models. A backward stepwise method was applied, and models were adjusted for potential confounders.

## 3. Results

A total of 260 patients were included in our study. Clinicopathological features of our cases are presented in Table 1. The mean age was 72 years and 55.8% of patients were men. Most patients were symptomatic (91.8%; systemic symptoms, 58%; local symptoms, 64.3%). The mean size and the depth of GC at diagnosis were 47.5 mm and 10.6 mm, respectively. According to the Borrmann classification, lesions were polypoid (type I, 18.7%), fungating (type II, 39%), ulcerated (type III, 32.4%) and flat/infiltrative (type IV, 10%). Most tumors were located in the gastric antrum (54.4%) or body (36.2%). A total of 67.5% of GC showed lymph node metastases at diagnosis. Tumors were stage I (14.3%), stage II (35.7%) and stage III (50%). Depending on the Laurén classification, GC was intestinal (58%), diffuse (32.7%) and mixed (9.3%). The percentage of tumors that were high grade was 52.8%, and 42.3% and 43.5% of cases showed lymphovascular invasion and perineural infiltration, respectively. Infiltrative growth pattern, desmoplasia, budding and tumor necrosis were observed in 64.1%, 49.7%, 23.4% and 24.6% of tumors, respectively. 31.7% of GC presented microsatellite instability (MSI). In total, 20% of patients received adjuvant therapy. During follow-up, 41.7% of tumors recurred (mean DFS: 40 months) and 30.4% of patients died due to GC (mean OS: 45 months).

### 3.1. Clinicopathological and Molecular Features Associated with Borrmann Types

Univariate analysis (Table 2) showed a significant association between Borrmann types and age at diagnosis, systemic symptoms, tumor size, Laurén subtype, presence of signet-ring cells, infiltrative growth pattern, high grade, tumor necrosis, size of the largest lymph node metastasis, MSI, HERCEPTEST and molecular type. The relationship between patient sex, perineural infiltration and Borrmann types tended to be significant (*p* = 0.051 and 0.097, respectively).

Regarding age at diagnosis, flat tumors were diagnosed in younger patients (mean age: 62 years) than polypoid/ulcerated (70–71 years) or fungating (77 years) lesions. Systemic symptoms decreased in frequency from 71.4% to 38.9%, from type I to type IV tumors. Ulcerated lesions were smaller at diagnosis (mean size: 30 mm) than polypoid, fungating or flat lesions (48–53 mm). With respect to the Laurén subtype, 67–73% of polypoid and fungating tumors and 49% of ulcerated lesions were intestinal-type GC. Flat lesions were more frequently diffuse-type GC (67%). Mixed GC did not show important differences between Borrmann types, but it was more common in ulcerated tumors (15.8%). Signet-ring cells were more frequent in flat (62.5%) and ulcerated (43.6%) GC, and these lesions were mostly of high grade (79% and 61%, respectively). The frequency of GC with an infiltrative growth pattern increased from type I (45%) to type IV (90.9%) lesions. Tumor necrosis was infrequent, but it was more commonly seen in fungating GC (34%). The size of the largest lymph node metastases decreased from type I to type IV lesions (from 15 to 6 mm).

As for molecular features, microsatellite instability was not detected in flat tumors, whereas 35–38.2% of Borrmann types I–III GC were microsatellite unstable. HERCEPTEST positivity was higher in type I tumors. In a previous investigation, we divided patients with GC into molecular subgroups according to the ARCG classification, using IHC as a surrogate for genetic alterations: gross morphology was significantly related to the molecular categories of GC [16]. In the cohort of advanced GC cases included in the TMAs (*n* = 180), this correlation was still significant. Most polypoid and ulcerated tumors were stable-p53 not overexpressed (60% and 49.1%, respectively) or microsatellite unstable. Fungating lesions were mostly stable-p53 not overexpressed or stable-p53 overexpressed and showed the highest rate of E-cadherin negative tumors (11.9%). Flat lesions were stable-p53 not overexpressed (86.4%) or stable-p53 overexpressed (13.6%).

As previously mentioned, the association between perineural infiltration, patient sex and Borrmann type tended to be significant. Polypoid and fungating tumors occurred more commonly in male patients, and the frequency of perineural infiltration increased from type I to type IV tumors.

**Figure 1:**
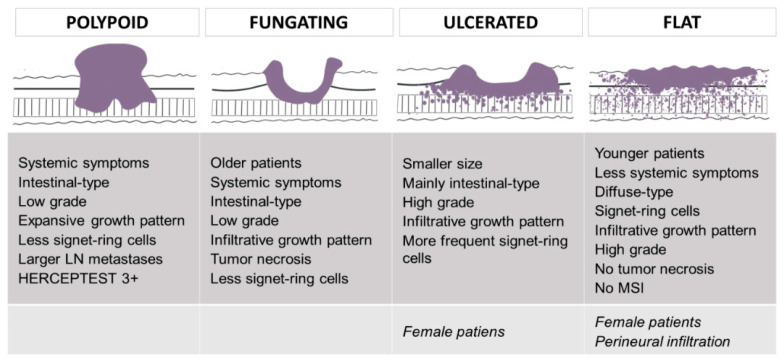
Main differential features of Borrmann types. Images designed by Alba Fernández Gutiérrez.

Figure 1 summarizes the main findings of the univariate analyses according to Borrmann type.

### 3.2. Prognostic Features

#### 3.2.1. Prognostic Features of Our Cohort

The univariate analysis is summarized in Table 3. Tumor recurrence was significantly associated with the presence of signet-ring cells, diffuse histology, perineural infiltration, vascular invasion, T stage, lymph node involvement and TNM stage. The relationship between GC recurrence and histological grade tended to be significant.

Cancer-specific death was significantly related to the presence of signet-ring cells, diffuse tumors, vascular invasion, infiltrative growth pattern, absence of desmoplasia, T stage, lymph node involvement and TNM stage. The association between cancer-specific death and histological grade tended to be significant.

The multivariate analysis is presented in Table 4. Laurén subtype, TNM stage and vascular invasion were independently related to tumor recurrence; Laurén subtype and TNM stage were independent prognosticators of cancer-specific death.

#### 3.2.2. Prognostic Role of the Borrmann Classification

Notably, no relationship was observed between patient outcomes (recurrence, cancer-specific death, OS or DFS) and Borrmann classification (Table 5). There were no significant differences in patient prognosis between type IV and types I–III GC, or between types III–IV and types I–II GC. As previously mentioned, we did not find an association between Borrmann classification and prognostic features such as T stage, lymph node involvement or TNM stage (Supplementary Table S1).

**Figure 2:**
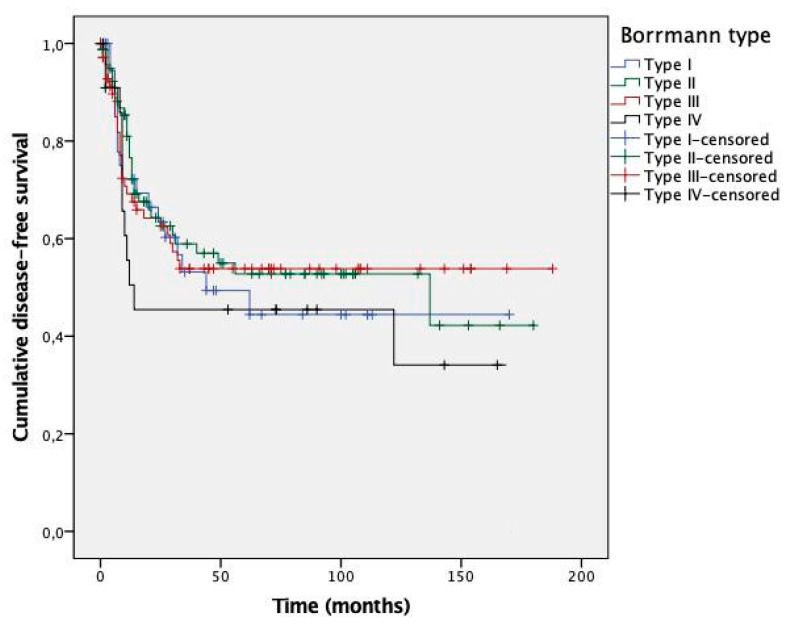
Kaplan–Meier curves for disease-free survival according to Borrmann type. The *p*-value by log-rank test was 0.607.

**Figure 3:**
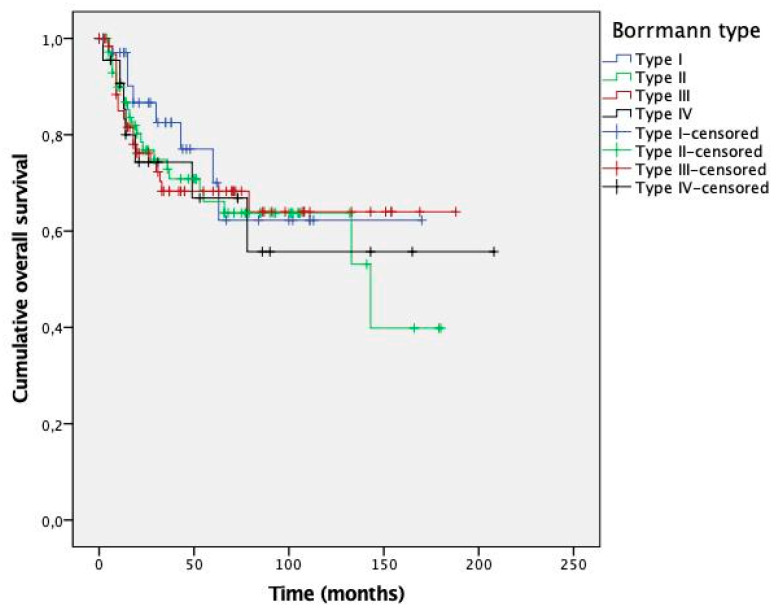
Kaplan–Meier curves for overall survival according to Borrmann type. The *p*-value by log-rank test was 0.895.

Kaplan–Meier curves are presented in Figure 2 (DFS) and Figure 3 (OS). No significant differences were observed between survival curves for different Borrmann types. Estimated survival times for patients with types I–IV GC are presented in Table 6.

## 4. Discussion

Several classifications of GC based on its gross appearance have been proposed, the most important being the Paris system, Borrmann classification and the Japanese classification of GC [13,17,18]. The Paris system divides early GC into three types and five subtypes. As previously mentioned, Borrmann classification applies to advanced GC and defines four categories. Finally, the Japanese classification includes both systems: the Paris system for superficial lesions (GC type 0), Borrmann classification for advanced lesions (types 1–4) and unclassifiable GC (type 5).

In this study, we found that Borrmann types were significantly related to different clinicopathological features: age at diagnosis, systemic symptoms, tumor size, Laurén subtype, presence of signet-ring cells, infiltrative growth pattern, high grade, tumor necrosis and size of the largest lymph node metastasis (Figure 1). Polypoid GC was associated with systemic symptoms, intestinal-type histology, low grade, expansive growth pattern and larger lymph node metastases. Fungating GC occurred in older patients with systemic symptoms, and it showed intestinal-type histology, low grade, infiltrative growth pattern and tumor necrosis. Signet-ring cells were rare in both types. Ulcerated lesions were related to smaller size at diagnosis, intestinal-type histology, high grade and infiltrative growth pattern. Signet-ring cells were more frequently observed. Finally, flat/infiltrative GC was diagnosed in younger patients with less systemic symptoms. Flat tumors were of the diffuse type and presented signet-ring cells, infiltrative growth patterns and high grade. Tumor necrosis was rare. Ulcerated and flat GC tended to occur in women.

In previous investigations, clinicopathological features of Borrmann types have been mainly assessed in type IV lesions. These tumors have been associated with features such as female sex, peritoneal involvement, younger age at diagnosis, larger size, high histological grade, lymph node metastases, tumor depth and higher stage at diagnosis [19,20,21]. A recent meta-analysis including 15 studies concluded that type IV GC showed high histological grade, lymph node metastases, peritoneal and serosal involvement, lymphatic invasion and poorer prognosis [22]. Furthermore, Zhou et al. demonstrated that abnormal expression of E-cadherin correlated with the diffuse-type GC and Borrmann types III and IV [23]. Notably, almost all of these investigations have been performed in Asian populations.

With respect to the molecular features of Borrmann types, Dai et al. analyzed HER2 positivity in Asian patients with advanced GC and observed that type III–IV tumors showed higher HER2 positivity than type I–II GC [23]. Interestingly, most cases of HER2-positive GC presented differentiated histology. We also found that HERCEPTEST positivity was more frequent in Borrmann types associated with intestinal or low-grade histology. In fact, HERCEPTEST was mainly positive in polypoid tumors (100% of 3+ cases were polypoid). We were not able to perform in situ hybridization (ISH) studies, but even if all HERCEPTEST 2+ cases turned out to be positive by ISH studies, the rate of HER2 positivity would still be higher in type I than in type II–IV tumors. On the other hand, we have not detected MSI in flat lesions, and the rate of MSI was similar across type I–III tumors (35–38.2%). Some authors have reported an association between MSI and Borrmann type II or I–II GC [24,25,26]. 

**Figure 4:**
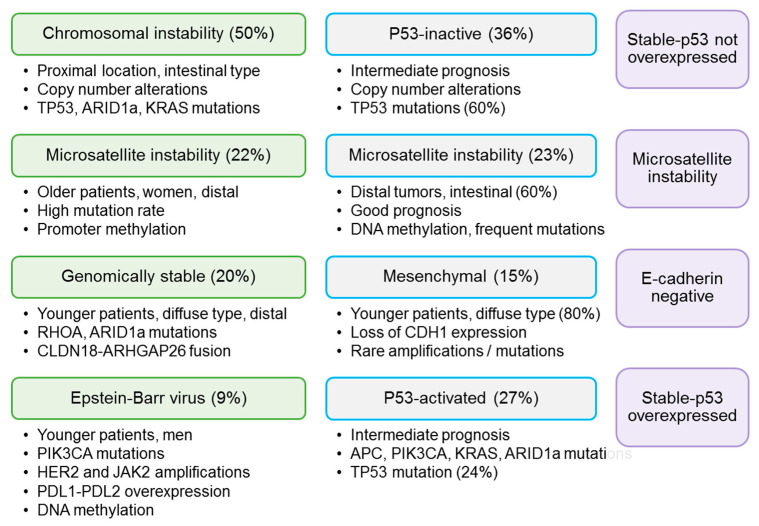
Molecular classifications of gastric cancer; from left to right: molecular subtypes as defined by The Cancer Genome Atlas Research Network, Asian Cancer Research Group and immunohistochemical-based classification.

In recent years, TCGA and the ACRG have developed important molecular classifications of GC [6,7]. TCGA described four types of GC: Epstein–Barr virus-positive GC, GC with microsatellite instability, genomically stable GC and GC with chromosomal instability (CIN) (Figure 4) [6]. The ACRG divided GC into GC with microsatellite instability, epithelial–mesenchymal transition GC, TP53 mutated GC and TP53 non-mutated GC, and these categories have been variably correlated with the molecular groups defined by TCGA (microsatellite instability, genomically stable, chromosomal instability and EBV-positive, respectively) [7]. Lai et al. analyzed the relationship between molecular groups (as defined by TCGA) and Borrmann classification, and observed that 70% of tumors with CIN were categorized as Borrmann types I–II, but the authors did not find a correlation between Borrmann classification and CIN status [27]. In a previous study, we divided a subset of GC patients from our institution into molecular subgroups according to the ARCG system, using IHC as a surrogate for genetic alterations (Figure 4), and we observed that gross morphology was significantly correlated with molecular categories [16]. This correlation was still significant in the cohort of patients with advanced GC. Polypoid and ulcerated tumors were mainly p53-not overexpressed or microsatellite unstable. Fungating tumors were mainly stable-p53 not overexpressed and stable-p53 overexpressed, and showed the highest rate of E-cadherin tumors. Finally, flat tumors were only stable-p53 not overexpressed (86.4%) or stable-p53 overexpressed (13.6%).

Regarding GC prognosis, previous investigations have variably related Borrmann classification to patient outcomes, mainly comparing type IV with type I–III GC. Thus, type IV GC has been identified as an independent prognostic factor in several studies [28,29]. Almost all of these studies have been conducted in Asian countries. Huang et al. analyzed 135 patients with Borrmann type IV GC and concluded that it should be classified as pT4b disease, because its prognosis was similar to that of pT4b tumors but poorer than pT2, pT3 and pT4a GC [30]. As for other Borrmann types, Kim et al. reported that type I GC is associated with higher recurrence rates than Borrmann types II and III, but there were no differences in the OS rate [31]. In our study, we did not detect a relationship between Borrmann types and prognosis. Furthermore, type IV tumors did not show significantly worse prognosis than type I–III GC. There are several possible explanations of our results. First, the number of patients may have been insufficient to achieve prognostic significance. However, we observed that multiple other clinicopathological features, such as Laurén subtype, vascular invasion and the TNM system, showed prognostic significance in our series (see Results section, Table 3), and multivariate analysis identified Laurén classification, TNM classification and vascular invasion as independent risk factors (see Results section, Table 4). Furthermore, Kaplan–Meier curves did not show a clear prognostic tendency for Borrmann types. Although type IV tumors showed slightly higher absolute recurrence and cancer-specific death rates, patients with type IV GC presented the highest mean OS. Furthermore, in some time periods, types I and III GC showed poorer prognosis than type IV tumors, as seen in the DFS and OS curves, respectively. Second, we established strict inclusion criteria and selected Western patients with advanced GC and curative R0 resections. Curative surgery may improve patient prognosis and decrease prognostic differences between Borrmann types. Third, GC incidence varies between geographical regions: 50% of all cases occur in Eastern Asia [32]. High incidence countries such as Korea or Japan have implemented screening programs, and survival rates are higher in these countries [33,34]. Previous research in Asian countries found that most patients with Borrmann type IV GC are diagnosed at advanced stages even in these areas; early diagnosis may significantly improve patient survival [19]. In our study, patients were not screened, most patients were symptomatic and most tumors were diagnosed at stages II–III. However, the TNM stage, T stage and lymph node involvement retained their prognostic impact, and we did not find significant differences between Borrmann types and T stage, lymph node involvement or TNM stage at diagnosis. According to these results, Borrmann classification may not show prognostic significance in Western patients with curative resections for GC. Further studies on Western patients should be conducted to address this issue.

With respect to GC treatment, some authors have suggested that type IV tumors may benefit less from surgical treatment [35,36], but other investigators have concluded that surgery significantly improves patient prognosis in type IV GC, regardless of tumor stage [37]. A recent meta-analysis confirmed that cases with non-curative resections had better prognosis than non-resected cases [22]. As previously stated, we did not find an association between Borrmann classification and prognosis, and all of our patients were treated by curative gastrectomy. Our findings support the notions that surgery may significantly improve patient outcomes in Borrmann type IV GC and that prognosis of patients with resected type IV GC may not be different from that for patients with resected types I–III GC.

## 5. Strengths and Limitations of Our Study

The results of this study should be interpreted in the context of its strengths and limitations. Strengths: We included patients with a curative gastrectomy and excluded cases with neoadjuvant therapy and R1–2 resections (homogeneous population). All patients were diagnosed in a Western tertiary center. All tumors were analyzed and pathological features were independently assessed by two pathologists, following a detailed protocol. Limitations: This study has a retrospective design. GC is not frequent in Western countries, so this study included a lower number of patients than Asian studies. Furthermore, the Spanish health system does not implement population-based screening for GC in the general population and most cases were symptomatic and diagnosed at stages II–III, with scarce representation of stage I GC in our series. IHC markers were performed in TMA sections and they may not represent the full heterogeneity of tumors. In an attempt to overcome this limitation, we selected cores from the center and the advancing front of the tumor. Furthermore, we did not observe significant differences between the two cores of each tumor.

## 6. Conclusions

In this study, we assessed the clinicopathological and molecular characteristics and the prognostic impact of Borrmann types in a cohort of patients with advanced GC from a Western institution. We observed a significant relationship between Borrmann types and several clinicopathological and molecular features of GC, including age at diagnosis, systemic symptoms, tumor size, Laurén subtype, presence of signet-ring cells, infiltrative growth pattern, high grade, tumor necrosis, HERCEPTEST positivity, MSI and molecular subtypes of GC.

Regarding patient outcomes, we identified Laurén classification, vascular invasion and TNM stage as independent risk factors for tumor recurrence and cancer-specific death. Previous investigations in Asian populations found that patients with type IV GC show significantly poorer prognosis than patients with types I–III GC. However, we did not find a relationship between Borrmann classification and prognosis. Further studies should analyze the prognostic significance of the Borrmann system in Western patients with curative resections for GC.

In summary, according to our results, Borrmann types show distinct clinicopathological and biological features, and Borrmann classification may serve as a cost-effective tool for patient stratification after GC resection. More studies in Western populations should be conducted in order to confirm the role of Borrmann classification in the classification of patients with advanced GC.

## Figures and Tables

**Table 1:** Clinicopathological features of our cases.

Feature		*n* (Valid %), *N* = 260 Mean (SD ^a^)
Age (years)		72 (12)
Men		156 (54.9%)
Smoking	Former	28 (10.9%)
	Ex-smoker	64 (24.8%)
Drinking	Former	24 (9.3%)
	Ex-drinker	11 (4.3%)
Symptoms	Symptomatic	191 (73.5%)
	Local	133 (64.3%)
	Systemic	120 (58%)
Size (mm)		47 (23)
Depth (mm)		10 (4)
Macroscopic type	Polypoid	45 (18.7%)
	Fungating	94 (39%)
	Ulcerated	78 (32.4%)
	Flat	24 (10%)
Location	Cardia	5 (2.1%)
	Fundus	17 (7.2%)
	Body	85 (36.2%)
	Antrum	128 (54.4%)
Tumor grade (high)		132 (52.8%)
Necrosis		64 (24.6%)
Vascular invasion		119 (42.3%)
Perineural infiltration		114 (43.9%)
Growth pattern (infiltrative)		134 (64.1%)
Budding		30 (23.4%)
Desmoplasia		98 (49.7%)
Intratumoral II ^b^	Mild	42 (20.5%)
	High	137 (66.8%)
Peritumoral II		65 65 (31.4%)
MSI		57 (31.7%)
HERCEPTEST	0	162 (92%)
	1+	7 (4%)
	2+	4 (2.3%)
	3+	3 (1.7%)
Molecular subtype ^c^	Type 1	45 (25.4%)
	Type 2	11 (6.2%)
	Type 3	90 (50.8%)
	Type 4	31 (17.5%)
T stage	T2	60 (23.1%)
	T3	152 (58.5%)
	T4	48 (18.5%)
N stage	N0	82 (32.5%)
	N1	48 (19%)
	N2	58 (23%)
	N3	64 (25.4%)
TNM stage	I	36 (14.3%)
	II	90 (35.7%)
	III	126 (50%)
Lymph node ratio		0.22 (0.27)
Gastrectomy	Subtotal	182 (71.4%)
	Total	73 (28.6%)
Lymphadenectomy	D1	33 (12.7%)
	D2	69 (26.5%)
	NS	156 (60.8%)
Adjuvant therapy		44 (20%)
Recurrence		100 (41.7%)
DFS ^d^ (mean, months, SD)		40 (46)
DFS (median, months)		16
Death		66 (30.4%)
Overall survival (mean, months, SD)		44 (45)
Overall survival (median, months)		27

^a^ SD: standard deviation; ^b^ II: inflammatory infiltration; ^c^ according to the Asian Cancer Research Group, as described in a previous study [16]. Type 1: microsatellite unstable; type 2: E-cadherin negative; type 3: stable-p53 not overexpressed; type 4: stable-p53 overexpressed. ^d^ DFS: disease-free survival.

**Table 2:** Univariate analysis. Clinicopathological features significantly related to Borrmann classification.

Feature (Valid %)		Polypoid (1)	Fungating (2)	Ulcerated (3)	Flat (4)	*p*
Age at diagnosis (years)		70	77	71	62	0.001
Systemic symptoms		71.40%	65%	49.20%	38.90%	0.032
Size (mm)		51.7	53.5	30	48.5	0.001
Laurén	Intestinal	66.70%	73.10%	48.70%	25%	<0.001
	Diffuse	31.10%	18.30%	35.50%	66.70%	
	Mixed	2.20%	8.60%	15.80%	8.30%	
Signet-ring cells		28.90%	25.80%	43.60%	62.50%	0.002
Infiltrative growth pattern		45%	65.30%	65.10%	90.90%	0.004
High grade		47.70%	37.50%	61.80%	79.20%	<0.001
Tumor necrosis		22.20%	34%	23%	0%	0.006
Largest LN ^a^ metastasis		15	11	9	6	0.002
MSI ^b^		35.10%	35%	38.20%	0%	0.016
HERCEPTEST	Negative	91.40%	98.30%	96.30%	95.50%	0.036
	2+	0%	1.70%	3.70%	4.50%	
	3+	8.60%	0%	0%	0%	
Molecular subtype ^c^	Type 1	31.40%	23.70%	34.50%	0%	<0.001
	Type 2	2.90%	11.90%	3.60%	0%	
	Type 3	60%	35.60%	49.10%	86.40%	
	Type 4	5.70%	28.80%	12.70%	13.60%	
*Sex (male)*		*60%*	*61.70%*	*41.60%*	*43.50%*	*0.051*
*Perineural infiltration*		*37.80%*	*40.40%*	*46.20%*	*66.70%*	*0.097*

^a^ LN: lymph node; ^b^ MSI: microsatellite instability; ^c^ according to the Asian Cancer Research Group, as described in a previous study [16]. Type 1: microsatellite unstable; type 2: E-cadherin negative; type 3: stable-p53 not overexpressed; type 4: stable-p53 overexpressed.

**Table 3:** Univariate analysis. Features significantly related to tumor recurrence and death.

Tumor Recurrence			
Feature		OR (95% CI)	*p*
Signet-ring cells		1.94 (1.14–3.32)	0.014
Laurén	Intestinal	1	0.004
subtype	Diffuse	2.59 (1.46–4.58)	
	Mixed	1.14 (0.45–2.92)	
Perineural infiltration		1.93 (1.14–3.24)	0.013
Vascular invasion		2.16 (1.28–3.66)	0.004
T stage	T2	1	0.012
	T3	2.09 (1.1–4.12)	
	T4	3.49 (1.49–8.2)	
Lymph node metastasis		2.96 (1.58–5.54)	<0.001
Lymph node ratio		0.19	
TNM stage	I	1	<0.001
	II	2.8 (1–8.03)	
	III	6.78 (2.45–18.81)	
*Tumor grade*		*1.61* (*0.96*–*2.72*)	*0.076*
**Cancer-specific death**			
**Feature**		**OR (95% CI)**	***p***
Signet-ring cells		1.96 (1.08–3.55)	0.026
Laurén	Intestinal	1	0.015
subtype	Diffuse	2.51 (1.33–4.72)	
	Mixed	1.58 (0.55–4.54)	
Vascular invasion		2.16 (1.2–3.88)	0.01
Growth pattern		2.52 (1.17–5.39)	0.016
Desmoplasia		0.48 (0.24–0.94)	0.032
T stage	T2	1	0.006
	T3	2.06 (0.92–4.65)	
	T4	4.54 (1.75–11.79)	
Lymph node metastasis		3.6 (1.7–7.63)	0.001
Lymph node ratio		<0.001	
TNM stage	I	1	<0.001
	II	4.34 (0.94–20.04)	
	III	10.44 (2.36–46.17)	
*Tumor grade*		*1.79* (*0.98*–*3.27*)	*0.058*

**Table 4:** Multivariate analysis. Independent risk factors for tumor recurrence and cancer-specific death.

Dependent Variable	Factor		*p*	HR (95% CI)
**Tumor recurrence**	Laurén subtype	Intestinal	0.016	
		Diffuse	0.008	1.796 (1.167–2.763)
		Mixed	0.790	0.901 (0.420–1.934)
	TNM stage	I	0.001	
		II	0.050	2.602 (1–6.767)
		III	0.001	4.627 (1.815–11.793)
	Vascular invasion		0.006	1.840 (1.195–2.835)
**Cancer-specific death**	Laurén subtype	Intestinal	0.022	
		Diffuse	0.007	2.453 (1.281–4.696)
		Mixed	0.615	1.297 (0.471–3.568)
	TNM stage	I	0.033	
		II	0.132	3.132 (0.709–13.823)
		III	0.022	5.416 (1.270–23.091)

**Table 5:** Univariate analysis. Relationship between Borrmann types, tumor recurrence and death.

	Type I	Type II	Type III	Type IV	*p*
Recurrence	42.2%	37.2%	39.7%	56%	0.411
Death	24.3%	31.8%	28.4%	32.4%	0.825

**Table 6:** Mean disease-free and overall survival times according to Borrmann type.

	Type I	Type II	Type III	Type IV
DFS ^a^ (mean, 95% CI ^b^)	87 (60–113)	98 (78–119)	106 (85–128)	76 (41–112)
OS ^c^ (mean, 95% CI)	120 (91–149)	113 (93–134)	128 (106–150)	131 (86–176)

^a^ DFS: disease-free survival; ^b^ CI: confidence interval; ^c^ OS: overall survival.

## Data Availability

Data are available on request.

## Supplementary Materials

The following are available online at https://www.mdpi.com/article/10.3390/cancers13123081/s1, Table S1: Correlation between Borrmann types and the TNM classification.
