# Supplementary material for: Are Borrmann’s Types of Advanced Gastric Cancer Distinct Clinicopathological and Molecular Entities? A Western Study
Source: Cancers (Basel). 2021 Jun 21;13(12):3081. doi: 10.3390/cancers13123081 (PMC8234739; doi:10.3390/cancers13123081)
Supplement: Supplementary file 1 [file cancers-13-03081-s001.zip › cancers-1268677-supplementary.pdf]

cancers MDPI

Table S1. Correlation between Borrmann types and the TNM classification.

		Type I	Type II	Type III	Type IV	p
**T**	T2	33.3%	20.2%	23.1%	16.7%	0.494
	T3	55.6%	59.6%	61.5%	58.3%	
	T4	11.1%	20.2%	15.3%	25%	
N+		64.4%	71.9%	65.3%	68.2%	0.770
TNM	I	17.8%	7.9%	19.2%	9.1%	0.345
	II	33.3%	41.6%	30.8%	31.8%	
	III	48.9%	50.6%	28.9%	59%	
